# COVID-19 as a social disease: qualitative analysis of COVID-19 prevention needs, impact of control measures and community responses among racialized/ethnic minorities in Antwerp, Belgium

**DOI:** 10.1186/s12939-022-01672-x

**Published:** 2022-05-16

**Authors:** Christiana Nöstlinger, Ella Van Landeghem, Jef Vanhamel, Anke Rotsaert, Lazare Manirankunda, Charles Ddungu, Thijs Reyniers, Deogratias Katsuva, Jana Vercruyssen, Stef Dielen, Marie Meudec

**Affiliations:** 1 Department of Public Health, Antwerp, Belgium; 2 Outbreak Research Team, Institute of Tropical Medicine, Antwerp, Belgium

**Keywords:** COVID-19, Community response, Immigrants, Racialized/ethnic minorities, Impact of control measures

## Abstract

**Background:**

In high income countries, racialized/ethnic minorities are disproportionally affected by COVID-19. Despite the established importance of community involvement in epidemic preparedness, we lack in-depth understanding of these communities’ experiences with and responses to COVID-19. We explored information and prevention needs, coping mechanisms with COVID-19 control measures and their impact on lived experiences among selected racialized/ethnic minority communities.

**Methods:**

This qualitative rapid assessment conducted in Antwerp/Belgium used an interpretative and participatory approach. We included migrant communities with geographic origins ranging from Sub-Saharan Africa, North-Africa to the Middle East, Orthodox Jewish communities and professional community workers. Data were collected between May 2020–May 2021 through key informant-, in-depth interviews and group discussions (*N* = 71). Transcripts were analyzed inductively, adopting a reflexive thematic approach. A community advisory board provided feedback throughout the research process.

**Results:**

Participants indicated the need for tailored information in terms of language and timing. At the start of the epidemic, they perceived official public health messages as insufficient to reach all community members. Information sources included non-mainstream (social) media and media from home countries, hampering a nuanced understanding of virus transmission mechanisms and local and national protection measures. Participants felt the measures’ most negative impact on their livelihoods (e.g. loss of income, disruption of social and immigration support). Economic insecurity triggered chronic stress and fears at individual and family level. High degrees of distrust in authorities and anticipated stigma were grounded in previously experienced racial and ethnic discrimination. Community-based initiatives mitigated this impact, ranging from disseminating translated and tailored information, providing individual support, and successfully reaching community members with complex needs (e.g. the elderly, digitally illiterate people, those with small social networks or irregular legal status).

**Conclusion:**

Study participants’ narratives showed how coping with and responding to COVID-19 was strongly intertwined with socio-economic and ethnic/racial characteristics. This justifies conceptualizing COVID-19 a social disease. At the same time, communities demonstrated resilience in responding to these structural vulnerabilities. From a health equity perspective, we provide concrete policy recommendations grounded in insights into communities’ structural vulnerabilities and resilience.

## Background

Increasing evidence in high income countries demonstrates that racialized/ethnic minority^1^ communities have been more severely impacted by COVID-19 than the general population [1–3]. They were over-represented in COVID-19 related hospitalizations in New York City [4], and in England among severe cases of COVID-19 [5]. Black people living in England and Wales were about four times more likely to die from COVID-19 compared with their white counterparts [6]. Explanations for this higher incidence and severity pertain to an interplay of factors at different levels [7, 8]. Lifestyle factors, genetic predisposition, or pathophysiological differences in infection susceptibility may interact with racial, socio-economic and other pre-existing health inequalities [9]. As with other infectious diseases such as HIV, malaria and tuberculosis [10], these conditions tend to cluster within socio-economically disadvantaged social groups, increasing their vulnerability for ill-health. Approaching COVID-19 as a social rather than simply an infectious disease is crucial to fully understand the multifaceted impact on individuals and communities [11].

COVID-19 and related non-pharmaceutical control measures have led to indirect consequences, both at population and individual level. For instance, stay-at-home measures and closure of businesses had adverse effects at economic, social and family levels further aggravating pre-existing health disparities [12, 13]. Furthermore, COVID-19 control measures are often not adapted to the working and living circumstances faced by many racialized/ethnic minority groups in high income countries. About 30% of immigrants in Europe live in poverty [14], hold low paid jobs or are engaged in informal economies facing a higher risk of losing their income [15]. In turn, living in crowded housing conditions may result in difficulties to comply with protection measures such as physical distancing [16] increasing COVID-19 exposure risk at individual [17] and community levels [9, 18]. In addition, irregular migration status often restricts access to health care services and testing facilities in many European countries [19]. COVID-19 and its control measures may thus amplify existing barriers to healthcare, contributing to increasing health inequities [20].

Belgium is a small country with about 11 million people living in densely populated (semi-)urban areas. Antwerp, a superdiverse city in Flanders has about 530,000 inhabitants of whom 50.4% were of foreign origin and 21.4% held foreign nationalities in 2020 [21]. About 92,000 persons are from Turkish and Maghreb descent, and 17,000 of sub-Saharan African (SSA) descent [22]. Among the estimated 25,000 Antwerp Jewish people, many do have the Belgian nationality. Some groupings – mainly the Orthodox Jewish communities – belong to larger networks of supranational communities or ‘courts’ [23, 24]. Antwerp neighborhoods with high concentrations of racialized/ethnic minorities are densely populated, ranging from 12,000–16,000 persons/km^2^ compared to about 6300 persons/km^2^ in the city center [25]. During the first wave of COVID-19, local media reported on “corona hotspots” when referring to high infection rates found in socio-economically deprived neighborhoods with high proportions of racialized/ethnic minorities [26, 27]. With more than 8800 additional deaths during the first wave, Belgium had a high COVID-19 related excess mortality. Elderly male immigrants had higher mortality rates in 2020 than natives compared to the previous year, with striking excess mortality rates for SSA women and men at middle age (40 and 70% increase respectively), typically related to their socio-economic and demographic position [28].

While a body of research demonstrates how social health determinants including migration can contribute to health inequities [29], the resources available to racialized/ethnic minority communities to cope with and react to an emerging health crisis have been less documented. Social networks, intergenerational cohesion and health supporting traditions are important health-promoting assets to respond to such crises [30]. Community involvement has shown to be a critical component in responding effectively to disease outbreaks [31], for instance during the Ebola epidemic in West-Africa [32]. Working with volunteers to provide community services has the potential to fill acute gaps and prevent public agencies from being overwhelmed during crisis events, such as COVID-19 [33]. Interdisciplinary, multilingual interventions led by community advocates reduced mistrust towards government-sanctioned medical programs in communities experiencing medical racism and health inequalities [34]. Ethnic/racialized minorities are not merely passive actors experiencing structural vulnerabilities, but they also respond actively to conditions and processes of vulnerabilization [35]. We lack in-depth insights into such dynamics, i.e. into how racialized/ethnic minorities in Antwerp have experienced COVID-19 and the related measures. This study’s objective was to explore COVID-19 related information and prevention needs including risk perceptions, the perceived impact of COVID-19, and community-based responses to COVID-19 among racialized/ethnic minority groups in Antwerp. In the present analysis, we focus on emerging transversal similarities across these communities while acknowledging their socio-economic, social and cultural heterogeneity.

## Methods

### Study background and design

This qualitative rapid assessment used an interpretative ethnographic participatory approach. We established a community advisory board (CAB) to enhance community participation throughout the entire research process, from providing advice on study objectives, through support with data collection, to joint interpretation of the findings.

### Study participants and sampling

Participants in this study were mainly from SSA, North-African and Middle-Eastern as well as from Orthodox Jewish communities (see Table 1). In recruiting participants, we started by including established community networks with community-based organizations (CBO) of SSA descent through an existing sexual health promotion program (i.e. the HIV-SAM project; see: www.hivsam.be). We focused on Orthodox Jewish, Moroccan, and Turkish communities due initial derogatory media reports framing them as being particularly involved in the spread of COVID-19. As participatory networks had yet to be set up with these communities, we addressed community representatives perceived to hold key positions in community networks. Using convenience and snowball sampling, we invited additional participants identified through these established structures. Key informants (KIs) included community leaders, health professionals, social workers with knowledge of these communities (often also native Belgians), and representatives of CBOs and faith-based organizations. For the interviews, we identified community members through collaborating CBOs. To assess potential changes as the pandemic evolved over time, we conducted additional key informant interviews approximately 1 year after the first lockdown (see data collection timelines below). Volunteers participating in a community-based information and awareness-raising initiative implemented by the City of Antwerp in socio-economically disadvantaged areas served as key informants in this second data collection round. They were thus were not identical with KIs interviewed during the first round of data collection. The selected groups were heterogeneous in terms of their ethnic descents and nationalities, socio-cultural, religious characteristics and immigration patterns. Thus, we adapted the research focus to the groups’ specific needs relevant to the overall objectives. Elsewhere, we have provided more details on the methodology, and the results on the Orthodox Jewish communities [36].

**Table 1:** Overview of study participants by data source, community, position and gender

	Participants (***N*** = 71) n (%)
**Data collection methods**	
Key informant interview (incl. 7 informal conversations)	49 (69)
In-depth interview	12 (17)
Group discussion (2 GD)	10 (14)
**Community belonging** ^a^	
Democratic Republic of the Congo	12 (17)
Belgium	12 (17)
Morocco	10 (14)
Orthodox Jewish	8 (11)
Secular/traditionally Jewish	5 (7)
Turkey	5 (7)
Nigeria	3 (4)
Burundi	2 (3)
Syria	2 (3)
Ghana	2 (3)
Cameroon	2 (3)
Uganda	2 (3)
South Africa	1 (1.5)
India	1 (1.5)
Guinea	1 (1.5)
Eritrea	1 (1.5)
Somalia	1 (1.5)
Sephardi	1 (1.5)
**Participant position**	
* Community participants*	
Community member (not affiliated to particular organizations)	22 (31)
Member of community organization	17 (24)
* Community workers*	
Social worker	12 (17)
Community health worker	5 (7)
Religious leader	4 (6)
Community leader	4 (6)
Civil servant	2 (3)
Immigration service worker	2 (3)
Health care worker	2 (3)
* Other:* Scientific expert	1 (1.5)
**Gender**	
Man	45 (63)
Woman	26 (37)

^a^Based on participants’ primary self-identification, may also include Belgian nationality

### Data collection

The first round of data collection took place between May–June 2020 (i.e. during the first lockdown) and the subsequent phase where some, but not all control measures were released (i.e. July–September 2020) during a resurgence of COVID-19 cases in certain neighborhoods followed by a local curfew in Antwerp in August 2020. The second round of data collection took place in May 2021, covering retrospectively the period November 2020 to May 2021, when Belgium had returned into lockdown again (i.e. 10/2020–04/2021) [37]. Trained qualitative social science researchers collected the data using the following data collection techniques, adapting them iteratively to respond to emerging topics:

KIs and *informal discussions* served to assess communities’ COVID-19 information and prevention needs, prevention responses and the impact of COVID-19 at community level. KIs were conducted with community leaders and professionals thought to have good insight and overview on how the epidemic evolved within their communities. They were interviewed on behalf of their communities. Informal conversations were shorter and less structured than KIs.

*Online group discussion* were conducted with community leaders and representatives to assess information and prevention needs, opinions and perceptions related to COVID-19 measures and the epidemic response, as well as recommendations for community-based responses.

*In-depth interviews* (IDIs) covered the lived experiences, individual coping strategies, as well as barriers and facilitators to prevention of individual community members.

Loosely structured open-ended topic guides were created for IDIs, KIs and group discussions. These topic guides enabled a natural flow of conversation and were adapted to the communities and data collection method. We ensured that the guides covered transversal core issues to enable comparison across participants and data sources. Core issues pertained to context-specific information on the respective communities; information on COVID-19, risk- and diseases perception; perception of prevention policies and control strategies, including their impact on communities. Data were collected mostly using online technologies, and in participants’ preferred language (English, Dutch, French, French-Lingala, French-Swahili). During the follow-up phase, all interviews but one were face-to-face.

### Data analytic approach

We analyzed the data inductively using NVivo 12.0 based on the verbatim transcribed interviews and for some, on extensive summaries. We adopted a reflexive thematic approach from a constructivist orientation [38]: After familiarization with the data, we performed the first coding in a semantic way, i.e. assigning codes to capture the explicit content by labeling important features. Next, we combined multiple codes to initial themes corresponding to the identified patterns of shared meaning. Several members of the research team then reviewed and refined the themes. With input and interpretation obtained from the CAB, we established final themes, each with their corresponding narratives.

## Results

Table 1 displays the characteristics of 71 study participants.

### Information sources, needs and barriers to accurate information

Participants’ narratives showed that most community members had good basic knowledge about COVID-19 and related prevention measures. Main information sources consisted of foreign television channels, information through family and friends, social media, and established community organizations. Some groups obtained information through media from their countries of origin rather than through official Belgian channels. This did not always reflect the local epidemic situation accurately, as illustrated by the following quote:

*"The crisis has shown that … a large group in the immigrant community also follows the news in the country of origin. ...mouth masks for instance, we have noticed that immigrants started using them much faster than others. As it was required there [Morocco]."* (KI, man, Moroccan community, civil servant)

Mainstream public health messages broadcasted in the national languages Dutch and French did not easily reach everyone. Participants mentioned both language and digital illiteracy as significant barriers:

*" … there is a great deal of people of foreign origin living in Belgium and in the beginning very little attention was paid to them. People assumed 'okay, everyone watches the news and they'll follow it all', but nothing could be further from the truth."* (KI, man, Turkish community, member of community organization)

The interviews and discussions showed that certain communities had difficulties in accessing nuanced information, such as newcomers with language problems, elderly first generation immigrants, and isolated or otherwise socially disadvantaged people. While these sub-groups reportedly were aware of the pandemic, they often could not keep up with the rapidly evolving scientific insights and acquire a nuanced understanding of transmission mechanisms and how this related to effective protection:

*"People understand 'I have to stay indoors', ‘I have to put on a mask’, but thinking about how this virus spreads and what makes me have to adapt my behavior to the current situation, is too difficult … . They can grasp direct guidelines, but the underlying idea of why those rules are there and how you can adjust your behavior to that, is difficult … some specific things are not getting through, like the coronavirus is in the air, not all these details were translated into other languages*." (GD participant, woman, Belgian, social worker)

In the beginning of the lockdown, translation of information through community initiatives took time. In addition, to respond to the needs of illiterate people of diverse foreign descent, prevention content had to be recorded and disseminated in audio format.

*"The government has written messages in all languages: Arabic, Hebrew, Syrian, … that's good.* *(* *… ) but not everyone can read their language, there are Turkish people who are orally Turkish, but they can't read it. So, we have been working with audios … ."* (KI, woman, Moroccan community, member of community organization)

Family and friends were also mentioned as important information sources, often updating the constantly changing information.

*"But, gosh. I would be surprised if people are not aware … Within families, there are children, young people, who often watch Flemish television and there are parents who watch Arabic or Turkish channels. But there is communication between the two, so I think that information usually reaches the parents.* (KI, man, Moroccan community, civil servant)

Participants agreed that people belonging to established communities or religious networks were reached quicker than those with small social networks, who lived in social isolation, who had no access to digital information or who were illiterate.

*"Most people are illiterate, so they ask other people ... and their church helps a lot in transferring information orally through announcements after services. The pastor did a lot at the beginning of the outbreak. He made a video and everyone got it … even translated in local languages the messages from government. ...People trust the church, because the way it is communicated."* (IDI, woman, Ghanaian church community)

The follow-up key informant interviews showed that during the third lock-down, questions about constantly changing regulations persisted, while concerns shifted to COVID-19 vaccination. Key informants reported that mistrust in the health care system and particularly in the vaccine was fueled by community members’ negative experiences with health care provision. A community health worker targeting Moroccan youth explained how social exclusion and lack of trust in societal institutions posed a continuous challenge to keeping young people well informed:

*“I tried to translate the measures to young people’s lingo, so that they knew what was allowed and what wasn’t...there is a lot of fake news online, and young people fall for it. They really believe that the vaccine renders girls infertile. Trust in the government and their policies is so damaged among these young people, not only because of corona, but of how they feel perceived by the society in general, especially those with migration background, they don’t feel that the government has their best interests at heart.”* (KI, woman, community health worker, Moroccan community)

Previous negative experiences and mistrust were also coupled with COVID-19 prevention fatigue:

*“People do not search for new information anymore, ... they don’t want even to look at it. People with low health literacy need to get information in a tailored, simple and slow way and they need to get opportunities to ask questions to a trusted source. But you will see, if someone poses questions to the staff at the reception, then the others will join in...”* (KI woman, social worker, Belgian descent)

### Risk perceptions

Most study participants perceived their communities to have about the same low risk of acquiring COVID-19 as the general population. At the beginning of the epidemic, people were said to have reacted with ignorance and denial, as shown by the following key-informant quote:

*“... in the beginning this pandemic hit us so suddenly and we didn’t accept it, so some people are still in denial. They didn’t believe that it can happen to us, that was very interesting to see. People were still talking in big groups...because they thought ‘no it will not hit us, I will not get corona’. They were saying things like it can only happen to old people or maybe people who do not have good health and background.“* (KI, man, community health worker, Indian descent)

At the start of the first lockdown, some people of SSA origin perceived their risk as even lower compared with the white majority population due to a combination of reasons, as exemplified here:

*“ … yeah, but … it’s only happening to the European or Caucasian people’. … but also because the numbers weren’t very high in Africa … and because they are new in the country, they still get a lot of information from Africa* … (IDI, woman, teacher at the integration classes, Somalian community)

Feeling personally affected emerged as an important motivational factor for adopting prevention behavior. Being cut-off from mainstream information deferred this process. For instance, religious practice prohibits the use of mainstream media and social networks for some Orthodox Jewish communities:

*"In the beginning everyone thought 'corona that's something in China, that's nothing for us'. Until it gets close... And if you have social media, and you follow mainstream media, it gets close faster. Because you see what's happening. If you don't have that, then you don't feel it until it really gets to yourself. Some Orthodox Jews didn't realize how close it already was until people they knew were infected." (IDI, man, Member of an Orthodox Jewish community)*

### Perceived negative impact of COVID-19

Several transversal themes emerged showing how the impact of COVID-19 was strongly intertwined with pre-existing socio-economic disadvantages.

#### Living circumstances

While the measures’ strongest effects were felt on income due to loss of job opportunities in informal economies, living circumstances were often described as extremely challenging for coping with the measures:

*"Many people from our community, about 80%, are disadvantaged people. Sitting with three, four children in an apartment without a garden … for them it has actually been enough. Especially in big cities... This has a lot to do with their social situation: housing, income, problematic family situations, problems, debts,... people have other priorities: finding food for their children, for example, everything else takes a back seat."* (KI, man, Moroccan community, social worker)

#### Socio-economic impact

Precarious working conditions were relatively common among study participants. Those working in uncertain conditions were not eligible to apply for temporary unemployment. Subsequently, many people kept on working whenever possible, even when experiencing symptoms of COVID-19:

*"... so some just kept working [...] but imagine they are coughing and they would have to stay at home for two weeks, that is a disaster for us. Therefore they often try not to cough. … Those people can't do without work and always try not to cough like that. "* (KI, man, Moroccan community, civil servant)

Many mentioned the bureaucratic complexities as a barrier to receive support, or needed help with the required administrative burden such as online submissions:

*“It was also difficult for them to ask for temporary unemployment … some of them did that too late .. and we organized support groups to fill the form and send it online … and others had their identity card expired … they got many problems, so they could not get their money at the right time.”* (KI, male, Community leader, Erithrean community)

People experiencing financial problems were also struggling to obtain prevention means such as mouth-masks, since only a limited number was disseminated for free by the City:

*"... mouth-masks and hand sanitizers, they cost a lot of money. Mouth-mask sold at the market were a lot cheaper, but of which quality are they? It can make a lot of difference. They [the government] should make sure that these people have all the prevention means at their disposal"* (IDI, man, community health worker, Belgian descent)

#### Psychosocial impact

For many participants, the pandemic created uncertainty towards the future, and the related measures triggered fears and chronic stress particularly among those who struggled to survive:

*“People have fear. They are worried about the future: will we still be able to live as we used to? They wonder if social life will survive... the pandemic takes all our time, we have lost our social habits. Also, there is the economic crisis, the prices in supermarket have increased, many are jobless because many companies have closed.”* (IDI, man, Cameroonian community)

The narratives also showed how COVID-19 impacted family dynamics and relationships. Dealing with future uncertainties, financial struggles, and being confined to small homes with large families was experienced as quite demanding:

*“ … the lockdown itself is very bad for people’s physical and mental well-being (...) There are also a lot of separations. I've had to do three appointments this week for a divorce, ..., it's been difficult for everyone. And you know, in our culture a man is always outside and all of a sudden he has to stay inside. Psychologically it was heavy, very heavy.* (KI, woman, Moroccan community, member of community organisation)

In addition, living with larger families – even when adhering to the social restrictions – could induce anticipated social stigma:

*“I am a father of thirteen children. We are all locked-up, we cannot even go to the park because my car only has seven seats. Even if we do go out, I see other people looking at us, ‘does he take that many people?’, but they do not understand, they are all part of my family*!” (IDI, male, Orthodox Jewish community member)

For some, anticipated stigma and the strict execution of the measures could lead to extreme social isolation. In particular those with limited access to updated information were said to be anxious and to isolate themselves more than needed, especially women and children:

*“I heard from several people who didn't come out for six, eight weeks. Nobody in the family except the husband for errands. Kids and moms mostly stayed inside.”* (GD participant, woman, social worker, Belgian)

However, other people felt targeted by the police and treated unfairly:

*"Police presence like this has never been seen before and people really do fear the fines, that's why they follow the measures very strictly, they also literally say: "I'm not going outside, I don't want to risk a fine because I can't pay it"*

(GD participant, woman, social worker, Belgian descent)

*" ...measures were policed differently and that is not quite right. I observed this, I went to the square X [in a middle-class neighborhood], there are many outdoor pubs and benches, I see a slightly whiter target group. Then go to square Y, [neighborhood with high proportion of foreign-born population) ]. You see people coming outside as well, they are different. A lot more young people and families with migrant background, but there are a lot more fines issued there than in the other neighborhood. That's not the only incident, those are stories from youth workers who have seen that for themselves."* (KI, man, Moroccan descent, social worker)

#### Disruption of social welfare services

During the first lockdown, social welfare services had discontinued or were only accessible online. The disruption of immigration and integration services, such as reception centers where people register for first asylum applications, hit asylum-seekers hard. Many others, for instance those with irregular migration status, were ineligible to obtain COVID-19 related support and they were cut off from support sources as well:

*“For migrants, especially those without papers, corona is a catastrophe; a threat to survival.”* (IDI , male, SSA communities)

The regulations’ perceived major impact on ethnic/racialized youth was restricted access to important support structures with both professionals and peers, according to the KIs. To mitigate the measures’ adverse effects, social workers at youth centers were thus asking for more flexibility to be able to continuously offer safe spaces for youth while respecting the safety measures:

*“Those young people don’t live in poverty, but they are vulnerable because of their cultural background, their networks, at school. They are often confronted with racism and discrimination. It’s already difficult for an adolescent to develop healthy, but these are additional barriers..’* (KI , woman, community health worker, Moroccan community)

For religious communities, the closing down of prayer-houses meant much more than not having the opportunity to pray, as shown by the following quote:

*"It [closing the synagogue] feels like something is missing, yes.... First of all because of the social aspects. I meet friends there every day. We have breakfast together, drink coffee before or after prayers and study the Talmud. Of course it feels like something is missing.... when you can't do what you've been doing three times a day for your whole life."* (IDI, man, community member of an Orthodox Jewish community)

### Community response and participation

*“So we did our best as much as we could and I think there is strength in that. We did this ourselves, we did not get it from above” (KI, man, Moroccan descent, social worker).*

Many bottom-up initiatives at grass-roots level emerged right at the beginning of the COVID-19 epidemic to mitigate the measures’ negative consequences. They ranged from countering the lack of information (e.g. translation services, WhatsApp messaging), to practical support (e.g. food aid, administrative support, referral services) to psychosocial support (delivered by phone, through informal support networks). Existing social networks and community ties such as churches and faith-based organizations were essential in reaching people, yet, they used dissemination tools such as social media platforms, which were new to them:

*“The mosque played an important role in communication to the communities, they have a special channel: a list of all members with data. These members can then be contacted and informed (...) in their own languages. Like, ‘This is the advice of the government and it must be followed’. Mosques have played a major role in the corona crisis, also through other channels such as Whatsapp*”. (KI, man, Moroccan descent, social worker)

Respondents reported to have missed policymakers’ recognition for their engagement. They also felt that authorities did not trust communities to apply measures correctly, yet flexibly, at the community level to continue offering much needed support services*.* They lacked consultation with policymakers before issuing or changing control measures:

*"We have no connections with the government, people are sitting at home, and it's really painful. If we had contact with the government ourselves, I'm sure we would have found a solution."* (IDI, man, member of an Orthodox Jewish community, religious community worker)

Community volunteers felt the need for more inclusive communication, as put by one volunteer:

*“It could be so much better. Involve your population and certainly the youth in decision-making. Sure, it's complicated to involve everyone, but there are already existing platforms and bodies that can be used to this end. They [the government] shouldn’t come up three days before the official communication of new measures so that everyone’s planning is messed up.”* (KI, woman, Moroccan community, social worker)

## Discussion

This study provides an in-depth understanding of how racialized/ethnic minorities experienced COVID-19 waves in 2020/21 in Antwerp. Narratives show an initial mismatch between public health information and communities’ needs of timely and tailored information on COVID-19 and related preventive measures. Official messages about the pandemic threat and the proposed control strategies were not conveyed in a way that allowed sufficient understanding of their rationale. The control measures likely exacerbated already existing socio-economic, ethnic-racial, and migration-related stressors that these communities face. Distrust in authorities and anticipated stigma were grounded in previous experiences of racial discrimination and actual observation of racial profiling at community-level.

We identified several cross-cutting vulnerability factors (e.g. language and digital barriers, living circumstances, reduced income, disrupted social services, etc.) that directly and indirectly influenced COVID-19 risk and related health burdens. Community responses, however, mitigated these effects as our data show. Community-based initiatives emerged early in the pandemic, based on existing community networks, but government recognition or support was not always perceived as adequate.

Our findings are in line with research showing that specific groups are particularly vulnerable to the wider health effects of the COVID-19 measures [12], and they highlight racialized/ethnic minority community members’ barriers to health care access [39]. Mainstream public health messages on COVID-19 were not adequately tailored to some immigrants and racial/ethnic minority communities, and did not consider vulnerability factors including language unproficiency [40]. Evidence shows indeed that urban districts with a population of lower economic and poorer health status and of non-Western descent had three-fold higher hospitalization rates during the first wave of COVID-19 compared to ethnic-Dutch individuals living in more affluent districts [41]. We found that many study participants over-relied on information channels from home countries and their informal social networks rather than on official national or local information channels, which is in line with research on linguistic barriers hindering their inclusion in public health efforts [42]. However, our findings also reveal the processes through which communities utilize resources to navigate the complexity of the information, such as translation and meaning-making within families through their children, trusted people and faith-based and community organizations.

Communities felt the measures’ strongest indirect effects on their income due to lost job opportunities, bureaucratic complexities forming a barrier to obtain COVID-19 related support or the impossibility to obtain this support (e.g. in the case of migrants with irregular status). Moreover, services set up to mitigate these disadvantages (e.g. social and immigration services) were disrupted due to the COVID-19 pandemic. Many immigrants work in low paid jobs in the informal economy, which infringes on their economic security. Intertwined with fluid conditions related to immigration trajectories this can lead to hyper-precarity, in which economic needs may prevail over health needs [42]. The current health crisis has highlighted these structural conditions, re-producing further health inequities through the indirect impact of COVID-19 control measures and shut down of services. For many, this has led to chronic stress and reduced mental well-being [42] with consequences at individual and family level. While psychological distress had increased for the overall Belgian population more than twofold compared with 2018, groups with pre-existing social inequalities ad lower access to social support experienced even higher levels of distress [43]. Recent research in Sweden among families living in socio-economically disadvantaged urban areas identified the pathways connected to health inequities. These ranged from low control over personal life situations through experiencing instability and insecurity, to having to care for children in poor and crowded housing conditions, struggling with reduced access to resources and limited social support [44].

At the community-level, the collective experiences of living in a segregated society was identified as another mechanism exacerbated by COVID-19. Many countries have seen (re) emergence of fear-based forms of discrimination in the margins of the COVID-19 epidemic, which are key-ingredients to racism and xenophobia [45]. The rise in antisemitic incidents reported in Belgium since the pandemic added to this evidence [46]. In this sense, our findings show how previous experiences of discrimination and the perceived unfairness in controlling the measures may have further weakened trust in public institutions. Trust has been characterized as a cognitive mechanism to reduce complexities [47], and its lack may lead to fears of being misled, skepticism towards control measures and false conspiracy theories. A survey conducted in England showed that such ideas were associated with lack of adherence to control measures and greater unwillingness to take up testing and treatment in the future [48]. Establishing trust among socio-economically disadvantaged people has been identified as an important precondition for successful screening, linkage to care and treatment, including contact tracing for COVID-19 [49, 50], and vaccine hesitancy [51]. Particularly for those most disadvantaged, such as people with irregular migration status, who have legitimate fears to get detained and deported [52, 53], trust and confidentiality in health-care provider relationships must be established.

### Importance of integrating and recognizing community responses

Many calls to action have been launched to address pre-existing health disparities that became visible during the COVID-19 pandemic [54, 55], but their implementation at community level lagged behind. Our research identifies two responses: first, the public health response took off slowly in these communities but gained momentum at the local level, as documented by initiatives such as training volunteers to raise awareness and increase adherence to the measures at community-level. Second, existing community networks, faith-based organizations and civil society filled the initial void and provided essential services to those in need. While this shows how resilient communities can be [56], these processes happened mostly in parallel to official responses without adequate planning, recognition, and resourcing. It has been recommended to engage these communities from the start of a pandemic outbreak [57] to provide necessary information accurately, in a timely manner to all members of society and to implement tailored interventions. In this sense, our research adds to documentation of good practices in community engagement [58]. In our context, it is especially relevant for trust-building given the institutional distrust found in our study. However, future research could investigate how such valuable ad-hoc responses could be translated into systematic community engagement in urban health governance.

### Limitations

This qualitative rapid assessment has several limitations. Data collection was restricted because of COVID-19-related regulations requiring us to collect data mostly online. We may have missed people with limited digital access or knowledge. Despite our efforts to achieve gender balance, we failed to do so. While this may partially reflect gender imbalances in organized community structures, it presents a selection bias. We collected data primarily among key informants acting as representatives of their respective communities, conducting comparatively fewer IDIs to directly elicit individual community members’ experiences. This may not always reflect individuals’ perceptions accurately. Because of the use of pre-existing community structures and our efforts to build new networks, we recognize that the data may not reflect the full heterogeneity of all racialized/ethnic minority groups residing in the study area. We aimed at learning from the perception of study participants’ with different ethnic descents to let similarities emerge despite their obvious historical, cultural and socio-economic differences. We also cannot rule out social desirability bias, as researchers interviewed the study participants mostly as outsiders. We mitigated these potential biases through careful triangulation of data sources and data collection methods and through consulting with the CAB three times during the research process in consultation meetings to increase the findings’ trustworthiness.

### Recommendations

Despite these limitations, the following policy and research recommendations emerged from the data and are applicable to the ongoing COVID-19 crisis and to potential similar future pandemics. *Viewing COVID-19 as an inherently social disease:* averting the economic, social and psychological consequences as described requires a health-in-all approach in the pandemic response [59, 60]. Thus, factors such as access to health care, housing, employment, food and spiritual support should be considered. Collaboration between health and non-health stakeholders and with strong integration of civil society building on communities’ resilience should be fostered.

*Integrating tailored COVID-19 responses through participatory approaches:* the need for diversified information on COVID-19 should be recognized and the communities targeted should be integrated into strategic public health planning. This provides the basis for linguistically and culturally sensitive messages, including prevention messages and tools for those who are (digitally) less literate. Participatory approaches can contribute to counter ethnic framing and avoiding stigmatization as shown by effective responses to HIV [61].

*Recognition of community responses*: our study shows that communities’ resilience enabled practical and quick solutions. Policymakers should therefore recognize community representatives as valuable and reliable partners, establish a planned division of labor, and equip them with adequate resources. Better representation of community members of racialized/ethnic minorities is needed at all levels of media, government, and health institutions to prevent further stigmatization of already marginalized groups through media coverage or government messages.

*Continuation of essential services:* avoiding the disruption of services from social welfare to immigration to psychosocial services is crucial to minimize the indirect impact of epidemic control measures [62]. Because of people’s economic precarity, a much faster adaptation of services to pandemic requirements is needed to maintain their accessibility.

*Establishing a data eco-system to produce disaggregated granular data:* Except for one study [28], no data on COVID-19 health outcomes linked to nationality or ethnic/racial criteria are available in Belgium. Along with research conducted elsewhere [17] studying the links between racial/ethnic characteristics and COVID-19 is an urgent research priority to better understand current gaps in health outcomes and address them.

## Conclusion

This study provides a snapshot of the evolving COVID-19 crisis from a community perspective. It shows that racialized/ethnic minorities’ perceptions of and coping strategies for COVID-19 have been strongly intertwined with socio-economic and structural vulnerability factors. The public health response to COVID-19 impacted the economic and psychosocial well-being of racialized/ethnic minorities, contributing to their increased vulnerabilization. This justifies the conceptualization of COVID-19 as a social disease. Our findings call for incorporating a health equity perspective into pandemic preparedness and response, building these insights into community response and participation.

## Data Availability

All relevant data supporting our findings are included in this published article. The complete datasets generated and analyzed during the current study are not publicly available because they might contain information that could identify confidential information relating to the study participants and other persons, yet additional pseudonymized data are available from the corresponding author on reasonable request.

## Footnotes

1 By *racialized* we refer to processes of racialization and categorization by which people are assigned a so-called “race”, emerging from the creation of hierarchies in social structures and systems. It is now widely accepted that “race” is a social construct. However, people have been racialized and then have subsequently shared common experiences of racism. By *ethnicity*, we refer to self-chosen identities encompassing characteristics such as language, nationality, culture and religion, allowing people to take on several identities.

## References

[1] Yaya S, Yeboah H, Charles CH, Otu A, Labonte R. Ethnic and racial disparities in COVID-19-related deaths: counting the trees, hiding the forest. BMJ Glob Heal. 2020;5(6):e002913. Available from: 10.1136/bmjgh-2020-002913. 10.1136/bmjgh-2020-002913 PMC7298686 32513864

[2] Aldridge RW, Lewer D, Katikireddi SV, Mathur R, Pathak N, Burns R (2020). Black, Asian and minority ethnic groups in England are at increased risk of death from COVID-19: indirect standardisation of NHS mortality data. Wellcome Open Res.

[3] van Dorn A, Cooney RE, Sabin ML. COVID-19 exacerbating inequalities in the US. Lancet [Internet]. 2020;395(10232):1243–4. Available from: 10.1016/S0140-6736(20)30893-X. 10.1016/S0140-6736(20)30893-X PMC7162639 32305087

[4] Wadhera R, Wadhera P, Prakriti G, Figueroa J, Joynt Maddox KE, Yey RW (2020). Variations in COVID-19 hospitalizations and deaths across New York City boroughs. JAMA.

[5] Williamson EJ, Walker AJ, Bhaskaran K, Bacon S, Bates C, Morton CE, et al. Factors associated with COVID-19-related death using OpenSAFELY. Nature [Internet]. 2020;584(7821):430–6. Available from: 10.1038/s41586-020-2521-4. 10.1038/s41586-020-2521-4 PMC7611074 32640463

[6] White C, Nafilyan V. Coronavirus (COVID-19) related deaths by ethnic group, England and Wales: 2 March 2020 to 15 May 2020. Off Natl Stat [Internet]. 2020;(April):1–10. Available from: https://www.ons.gov.uk/peoplepopulationandcommunity/birthsdeathsandmarriages/deaths/articles/coronaviruscovid19relateddeathsbyethnicgroupenglandandwales/2march2020to15may2020%0A. https://www.ons.gov.uk/peoplepopulationandcommunity/birthsdeathsandmarriages/d

[7] Millett GA, Jones AT, Benkeser D, Baral S, Mercer L, Beyrer C (2020). Assessing differential impacts of COVID-19 on black communities. Ann Epidemiol.

[8] Hooper W, Napoles AM, Perez-Stable E (2020). COVID-19 and racial/ethnic disparities. JAMA.

[9] Khunti K, Singh AK, Pareek M, Hanif W (2020). Is ethnicity linked to incidence or outcomes of covid-19?. BMJ.

[10] Velavan TP, Meyer CG, Esen M, Kremsner PG, Ntoumi F, Pandora-ID-Net (2021). COVID-19 and the syndemic challenges in battling the big three: HIV, TB and malaria. Int J Infect Dis.

[11] Horton R (2020). Offline: COVID-19 is not a pandemic. Lancet.

[12] Douglas M, Katikireddi SV, Taulbut M, McKee M, McCartney G (2020). Mitigating the wider health effects of covid-19 pandemic response. BMJ [Internet]..

[13] Greenaway C, Hargreaves S, Barkati S, Coyle CM, Gobbi F, Veizis A (2021). COVID-19: exposing and addressing health disparities among ethnic minorities and migrants. J Travel Med.

[14] OECD, European Commission. Settling In: OECD Indicators of Immigrant Integration 2012 [Internet]. Settling In: OECD Indicators of Immigrant Integration 2012. 2018. Available from: https://www.oecd-ilibrary.org/docserver/9789264307216-en.pdf?expires=1638282319&id=id&accname=guest&checksum=771B5DD7D5EEE82FA67F0358FA9A57F3

[15] International Organization of Labor. COVID-19 crisis and the informal economy [Internet]. Vol. 2015, ILO Brief 2020. Available from: https://www.ilo.org/global/topics/employment-promotion/informal-economy/publications/WCMS_743623/lang%2D%2Den/index.htm

[16] Capolongo S, Rebecchi A, Buffoli M, Appolloni L, Signorelli C, Fara GM (2020). COVID-19 and cities: from urban health strategies to the pandemic challenge. A decalogue of public health opportunities. Acta Biomed.

[17] Bhala N, Curry G, Martineau AR, Agyemang C, Bhopal R (2020). Sharpening the global focus on ethnicity and race in the time of COVID-19. Lancet..

[18] Yancy CW (2020). COVID-19 and African Americans. JAMA - J Am Med Assoc..

[19] Carillon S, Gosselin A, Coulibaly K, Ridde V, Desgrées du Loû A (2020). Immigrants facing Covid 19 containment in France : an ordinary hardship of disaffiliation. J Migr Heal.

[20] Germain S, Yong A (2020). COVID-19 highlighting inequalities in access to healthcare in England: a case study of ethnic minority and migrant women. Fem Leg Stud.

[21] Vlaanderen. S. Lokale inburgerings- en integratiemonitor – Antwerpen. [Internet]. 2020. Available from: https://integratiebeleid.vlaanderen.be/sites/default/files/atoms/files/2020_Vlaamse-LIIM_Antwerpen_0.pdf%0A

[22] Loos J, Nöstlinger C, Vuylsteke B, Deblonde J, Ndungu M, Kint I, et al. First HIV prevalence estimates of a representative sample of adult sub-Saharan African migrants in a European city. Results of a community-based, cross-sectional study in Antwerp, Belgium. PLoS One. 2017;12(4). Available from: 10.1371/journal.pone.0174677. 10.1371/journal.pone.0174677 PMC5381894 28380051

[23] Schnitzer G, Loots G, Escudero V, Schechter I (2011). Negotiating the pathways into care in a globalizing world: help-seeking behaviour of ultra-orthodox Jewish parentsNo title. Int J Soc Psychiatry.

[24] Abicht L. De Joden van Antwerpen. 1st ed. Antwerpen: Vrijdag Uitgevers; 2018.

[25] Stad Antwerpen. De stad in cijfers. [Internet]. 2021. Available from: https://stadincijfers.antwerpen.be/dashboard/hoofd-dashboard/demografie/No Title.

[26] Bervoet D, Segers T. Borgerhout is coronahotspot in Antwerpen. De Tijd. 2020;31. Available from: https://www.tijd.be/dossiers/coronavirus/borgerhout-is-coronahotspot-in-antwerpen/10242409.html.

[27] Willocx C. Kwart van besmettingen komt uit Borgerhout: “Het was te laat voor ingrepen op wijkniveau”. Gazet van Antwerpen. 2020;31. Available from: https://www.gva.be/cnt/dmf20200731_97243976.

[28] Vanthomme K, Gadeyne S, Lusyne P, Vandenheede H (2021). A population-based study on mortality among Belgian immigrants during the first COVID-19 wave in Belgium. Can demographic and socioeconomic indicators explain differential mortality? SSM. Popul Heal.

[29] Castañeda H, Holmes SM, Madrigal DS, Young MEDT, Beyeler N, Quesada J (2015). Immigration as a social determinant of health. Annu Rev Public Health.

[30] WHO regional Office for Europe. How health systems can address health inequities linked to migration and ethnicity. WHO Reg Off Eur. 2010. https://www.euro.who.int/__data/assets/pdf_file/0005/127526/e94497.pdf.

[31] Marston C, Renedo A, Miles S (2020). Community participation is crucial in a pandemic. Lancet..

[32] Bedson J, Jalloh MF, Pedi D, Bah S, Owen K, Oniba A (2020). Community engagement in outbreak response: lessons from the 2014-2016 Ebola outbreak in Sierra Leone. BMJ Glob Heal.

[33] Miao Q, Schwarz S, Schwarz G. Responding to COVID-19: Community volunteerism and coproduction in China. World Dev. 2021;137:105128. Available from: 10.1016/j.worlddev.2020.105128. 10.1016/j.worlddev.2020.105128 PMC7413054 32834397

[34] Chamie G, Marquez C, Crawford E, Peng J, Petersen M, Schwab D (2021). Community transmission of severe acute respiratory syndrome coronavirus 2 disproportionately affects the latinx population during shelter-in-place in San Francisco. Clin Infect Dis.

[35] McLaren L, Masuda J, Smylie J, Zarowsky C. Unpacking vulnerability: towards language that advances understanding and resolution of social inequities in public health. Can J Public Heal 2020;111(1):1–3. Available from: 10.17269/s41997-019-00288-z. 10.17269/s41997-019-00288-z PMC7046829 31994015

[36] Vanhamel J, Meudec M, Van Landeghem E, Ronse M, Gryseels C, Reyniers T (2021). Understanding how communities respond to COVID-19: experiences from the orthodox Jewish communities of Antwerp city. Int J Equity Health.

[37] Luyten J, Schokkaert E. Belgium’s response to the Covid-19 pandemic. Heal Econ Policy Law. 2021:1–11. Available from: 10.1017/S1744133121000232. 10.1017/S1744133121000232 PMC8280466 34219632

[38] Braun V, Clarke V (2019). Reflecting on reflexive thematic analysis. Qual Res Sport Exerc Heal.

[39] Hooper MW, Mitchell C, Marshall VJ, Cheatham C, Austin K, Sanders K, et al. Understanding multilevel factors related to urban community trust in healthcare and research. Int J Environ Res Public Health. 2019;16(18). Available from: 10.3390/ijerph16183280. 10.3390/ijerph16183280 PMC6765868 31500126

[40] Shadmi E, Chen Y, Dourado I, Faran-Perach I, Furler J, Hangoma P (2020). Health equity and COVID-19: global perspectives. Int J Equity Health.

[41] Coyer L, Wynberg E, Buster M, Wijffels C, Prins M, Schreijer A (2021). Hospitalisation rates differed by city district and ethnicity during the first wave of COVID-19 in Amsterdam, The Netherlands. BMC Public Health.

[42] Guadago L. Migrants and the COVID-19 pandemic : an initial analysis [internet]. International Organization of Migration. Migration Research Series. 2020; Available from: Lorenzo. Available from: https://publications.iom.int/books/mrs-no-60-migrants-and-covid-19-pandemic-initial-analysis.

[43] Lorant V, Smith P, Van den Broeck K, Nicaise P (2021). Psychological distress associated with the COVID-19 pandemic and suppression measures during the first wave in Belgium. BMC Psychiatry.

[44] Barboza M, Marttila A, Burström B, Kulane A (2021). Covid-19 and pathways to health inequities for families in a socioeconomically disadvantaged area of Sweden – qualitative analysis of home visitors’ observations. Int J Equity Health.

[45] Devakumar D, Shannon G, Bhopal SS, Abubakar I. Racism and discrimination in COVID-19 responses [internet]. Lancet. 2020;395. 10.1016/S0140-6736(20)30792-3. 10.1016/S0140-6736(20)30792-3 PMC7146645 32246915

[46] Tel Aviv University. Antisemitism Worldwide 2011. 2011;1–94. Available from: http://kantorcenter.tau.ac.il/event/antisemitism-worldwide-2011.

[47] Fonagy P, Luyten P, Allison E, Campbell C (2017). What we have changed our minds about: part 1. Borderline personality disorder as a limitation of resilience. Borderline Personal Disord Emot Dysregulation.

[48] Freeman D, Waite F, Rosebrock L, Petit A, Causier C, East A, et al. Coronavirus conspiracy beliefs, mistrust, and compliance with government guidelines in England. Psychol Med. 2022;52(2):251–63. Available from: 10.1017/S0033291720001890. 10.1017/S0033291720001890 PMC7264452 32436485

[49] Fields VL, Kiphibane T, Eason JT, Hafoka SF, Lopez AS, Schwartz A (2020). Assessment of contact tracing for COVID-19 among people experiencing homelessness, salt Lake County health department,March–May 2020.

[50] Langellier BA. Policy recommendations to address high risk of COVID-19 among immigrants. Am J Public Health. 2020;110(8):1137–9. Available from: 10.2105/AJPH.2020.305792. 10.2105/AJPH.2020.305792 PMC7349431 32584591

[51] Europe WHO. Vaccination and trust [internet]. World Health Organization Regional office for Europe. 2017; Available from: http://www.euro.who.int/en/health-topics/disease-prevention/vaccines-and-immunization/publications/2017/vaccination-and-trust-2017.

[52] Van Durme C. “Firewall”: a tool for safeguarding fundamental rights of undocumented migrants. [internet]. Platform for International Cooperation on Undocumented Migrants. Available from: https://picum.org/firewall-tool-safeguarding-fundamental-rights-undocumented-migrants/.

[53] Beck TL, Le TK, Henry-Okafor Q, Sha MK (2020). M e d i c a l C a re fo r undocumented immigrants national and international issues. Prim Care Clin Off Pract.

[54] Laurencin CT, McClinton A (2020). The COVID-19 pandemic: a call to action to identify and address racial and ethnic disparities. J Racial Ethn Heal Disparities.

[55] Orcutt M, Patel P, Burns R, Hiam L, Aldridge R, Devakumar D (2020). Global call to action for inclusion of migrants and refugees in the COVID-19 response. Lancet.

[56] Jewett RL, Mah SM, Howell N, Larsen MM (2021). Social cohesion and community resilience during COVID-19 and pandemics: a rapid scoping review to inform the United Nations research roadmap for COVID-19 recovery. Int J Health Serv.

[57] Kickbusch I, Reddy SK (2016). Community matters– why outbreak responses need to integrate health promotion. Glob Health Promot.

[58] Gilmore B, Ndejjo R, Tchetchia A, De Claro V, Mago E, Diallo AA (2020). Community engagement for COVID-19 prevention and control: a rapid evidence synthesis. BMJ Glob Heal..

[59] Bucciardini R, Contoli B, De Castro P, Donfrancesco C, Falzano L, Ferrelli R (2020). The health equity in all policies (HEiAP) approach before and beyond the Covid-19 pandemic in the Italian context. Int J Equity Health.

[60] Trout LJ, Kleinman A (2020). Covid-19 Requires a Social Medicine Response. Front Sociol.

[61] Logie CH (2020). Lessons learned from HIV can inform our approach to COVID-19 stigma. J Int AIDS Soc.

[62] United Nations network on migration. Enhancing access to Services for Migrants in the context of COVID-19 preparedness, Prevention, and Response and Beyond [Internet] 2020. Available from: https://www.un.org/en/ga/search/view_doc.asp?symbol=A/RES/73/195

